# Supplementary material for: Degradation Mechanism of Aflatoxin M1 by Recombinant Catalase from Bacillus pumilusE-1-1-1: Food Applications in Milk and Beer
Source: Foods. 2024 Mar 15;13(6):888. doi: 10.3390/foods13060888 (PMC10969422; doi:10.3390/foods13060888)
Supplement: Supplementary file 1 [file foods-13-00888-s001.zip › foods-2629855-supplementary.pdf]

Supplementary File

S1. CAT concentration and activity

The reaction solution contained CAT assay working solution and 1 U rCAT, mixed for 5 s. The initial absorbance value of the working solution was determined at 240 nm, called A_1, and the absorbance value after 1 min at room temperature was called A_2. The enzyme activity of rCAT was calculated according to the following equation (Solarbio Life Science, Beijing, China, BC0200).

$$CAT\ (U / mL) = 678 * (A_1 - A_2) / Cpr$$

Cpr is the concentration of supernatant protein.

S2. Measurement of AFM_1 concentrations by HPLC

High-performance liquid chromatography (HPLC) was performed using an Agilent (California, USA) separation unit and an Agilent (LC-20 AT, Agilent, USA) fluorescence detector (λ_{ex} = 360 nm, λ_{em} = 410 nm). The HPLC method for AFM_1 analyses was as follows: the chromatographic column was a Diamonsil® C18 column (5 μm; 250 mm × 4.6 mm; Supelco Company, Bellefonte, USA). The mobile phase was acetonitrile: water (25:75, v/v), with a flow rate of 1 mL/min at a column temperature of 30 °C.

A standard curve was prepared with five different concentrations of AFM_1 solutions in the concentration range of 25-400 ng/mL (25, 50, 100, 200, and 400 ng/mL). The holding time and calibration curve for each standard were both used to detect AFM_1 and its concentration in samples.

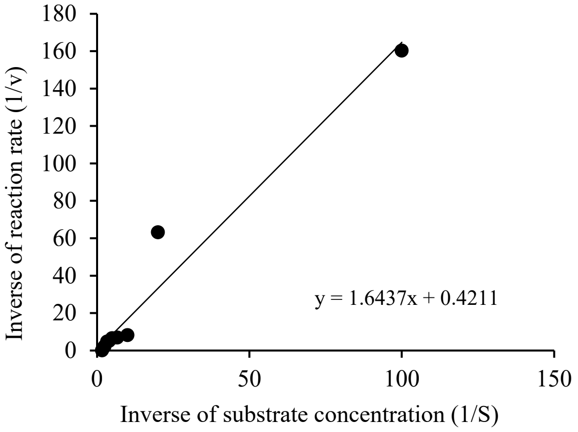

Figure S1. Enzymatic reactions as indicated by a Lineweaver–Burk diagram

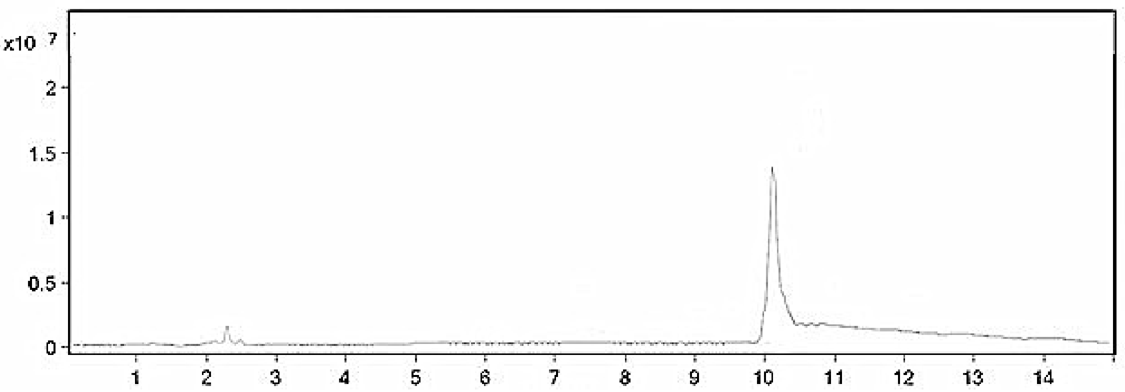

Figure S2. Total ion flow diagram of the AFM_1 standard solution in positive ion mode

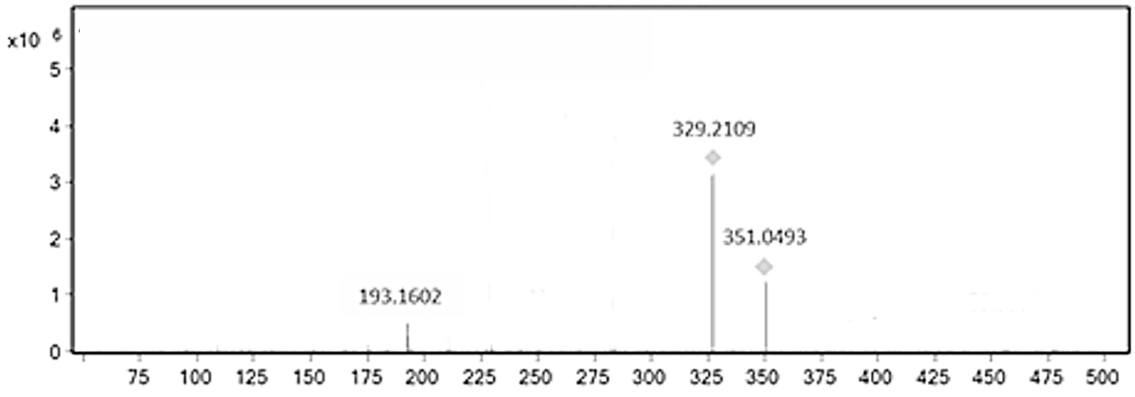

Figure S3. Primary mass spectrometry of the AFM_1 standard solution in positive ion mode

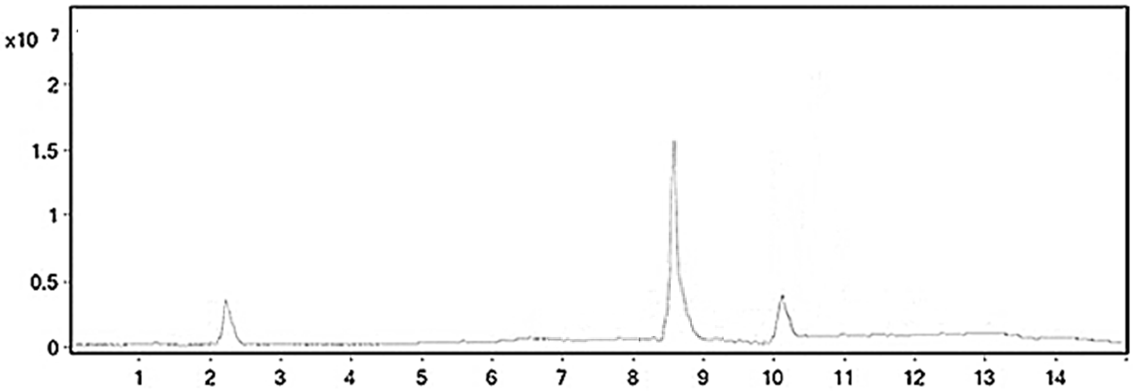

Figure S4. Total ion flow diagram of degradation products produced by rCAT treatment in positive ion mode

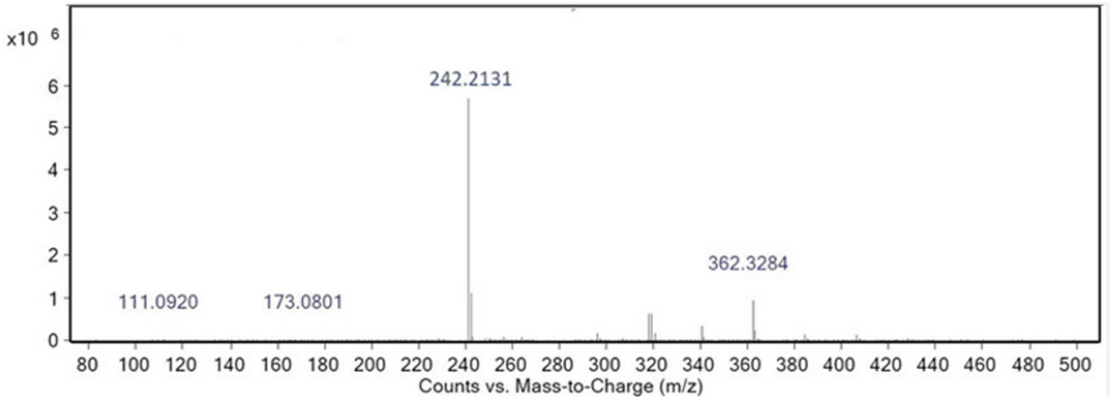

Figure S5. Primary mass spectrometry of degradation products produced by rCAT treatment in positive ion mode
